# Establishing priorities for diabetes action goals according to key opinion leaders and health professionals

**DOI:** 10.1186/s13584-022-00540-x

**Published:** 2022-08-19

**Authors:** Dana Zelnik Yovel, Orly Tamir, Elza Lavon, Tanya Kolobov, Anat Bel-Ange, Michal Julius, Itamar Raz, Micha Rapoport

**Affiliations:** 1 Department of Internal Medicine “C”, Shamir Medical Center, Zerifin, Israel; 2 Pesach Segal Israeli Center for Diabetes Research and Policy, Sheba Medical Center, Ramat Gan, Israel; 3 National Diabetes Council, Jerusalem, Israel; 4 Ministry of Health, Jerusalem, Israel; 5 Shamir Medical Center, Endocrinology Institute, Zerifin, Israel; 6 Diabetes Medical Center, Tel Aviv, Israel

**Keywords:** Diabetes mellitus, National diabetes program, Priorities, Policy

## Abstract

**Background:**

The ever-increasing burden of diabetes and the limited resources highlight the need for prioritization of national action goals for diabetes management. The Israeli National Diabetes Council (INDC) initiated a prioritization process aiming to set a top list of diabetes related goals, as suggested by decision makers and health professionals.

**Methods:**

A 2-step prioritization process, including a small (n = 32) circle of key opinion leaders of the INDC and a larger (n = 195) nationwide circle of diabetes health professionals consisting of physicians, nurses, and dieticians working in diabetes care centers, hospitals and family practice clinics, was established. An online questionnaire presenting 45 different action areas in diabetes prevention and care was distributed to the INDC members who ranked the 3 top diabetes priorities based on their individual interpretation of importance and applicability. The 7 highest ranking priorities were later presented to hospital-based and community diabetes health professionals. These professionals selected the 3 top priorities, based on their perceived importance.

**Results:**

Council members opted mostly for action areas regarding specific populations, such as clinics for adult type-1 diabetes patients, diabetic foot, and pediatric and adolescent patients, while the health professionals’ top priorities were mostly in the general field of prevention, namely high-risk prediabetes population, prevention of obesity, and promotion of healthy life-style. In addition, priorities differed between hospital and community health professionals as well as between different professional groups.

**Conclusions:**

A national prioritization process of action areas in diabetes prevention and care is attainable. The resulting item list is affected by professional considerations. These priorities may direct efforts in the implementation of interventions to improve national-level diabetes management.

**Supplementary Information:**

The online version contains supplementary material available at 10.1186/s13584-022-00540-x.

## Background

The estimated total economic cost of diagnosed diabetes is ever increasing reaching in 2030 an estimated global expenditure of $2.5 trillion worldwide [1, 2]. This highlights the substantial burden that diabetes imposes on most societies and the resulting crucial need for optimal utilization of resources and prioritization of diabetes treatment and prevention strategies. The aim of prioritization is setting a top list of action goals based on the action goals’ importance and applicability in a given national socioeconomic and healthcare environment and should reflect the integrated values of the society and the local attitude to health management. Thus, the question is not whether to set priorities, but how to improve prioritization processes.

Such a prioritization process might be affected by multiple factors such as socioeconomic status, medical specialty, individual values and preferences, as well as the general health management policy. Previous studies regarding diabetes-related prioritization had focused on diabetes research [3, 4] while others examined the goals of individual patients [5, 6] or, guideline development [7, 8]. Other studies examined cost-effectiveness of various interventions such as prevention of diabetes in high-risk individuals [9]. However, there is no available data regarding an effort of setting a top list of priorities for diabetes management on a national level.

The Israeli National Diabetes Council (INDC) is one of 22 national councils, which are professional multi-system bodies that provide advice to the directors of the Ministry of Health (MoH) and policy makers of the health care system regarding the various fields of medicine. The council deals with issues relevant to heterogeneous populations, and addresses conceptual, organizational, medical and logistic aspects related to provision of healthcare within the system. The INDC is comprised of all stakeholders relevant to diabetes, including representatives of the health organizations in the country, the MoH, various medical professions and patient's advocates. In 2012 the council was assigned the task of developing and direction of a National Program for Diabetes Prevention and Care. This program was developed between 2012 and 2014 and officially launched in 2016 [10]. It was divided into 8 strategies, which were further subdivided into particular action areas, encompassing a total of 45 different topics.

The main goal of our study was to highlight the most important topic having the highest potential for implementation, by mapping the top priorities suggested by both key diabetes leaders and health professionals in Israel.

## Material and methods

### Study design & participants

This study was conducted in two stages:

(A) The INDC members selected seven top priorities from the 45 action areas listed in the National Diabetes Program.
(B) A wide range of diabetes care professionals selected the three top-priority action areas from the list of seven priorities created in the previous step.

#### Step A—Prioritization by the INDC members

An online questionnaire containing 45 different action areas in diabetes prevention and care (Additional file 1) was distributed in June 2020 to all 38 members of the INDC using the Google form online tool. A reminder was sent via email and SMS a week later.

Each participant was asked to provide his personal demographic and professional details. Participants chose the 3 top important priorities and then were asked to grade each one’s applicability on a scale from 1 to 5, according to their subjective opinion (1- the least applicable, 5- the most applicable). The most important topic was assigned a score of 3, second ranked topic was assigned a score of 2 and the third a score of 1. For each topic a final score was calculated by multiplying the importance score by mean applicability. The seven topics that received the highest final score were used for step B. In order to validate our strategy to integrate both importance and applicability, we conducted an internal comparison of two lists: one created by integrating both importance and applicability and the second with calculation of importance alone. Rank of the topics in each list was very much similar but not identical (R = 0.97, *P* < 0.001), which suggests that each has a unique contribution to the final score, hence supporting our methodology.

#### Step B—Prioritization by hospital and community diabetes health professionals

The list of seven topics from step A was presented online to various Israeli diabetes health professionals. These included primary care physicians, internal medicine specialists, endocrinologist, diabetes educators, diabetes nurse specialists and diabetes-oriented dieticians, working in community primary care, diabetes specialized clinics, and medical centers. Each responder was asked to select the three most important priorities out of the seven. Applicability was not addressed, assuming that health professionals have less insight in that regard compared to INDC members. Each responder was also asked to provide his main professional occupation, demographic information, main work place (community/medical center) and geographic location.

### Statistical analysis

Descriptive statistics were used to describe background information of the responders. Categorical variables were reported as frequencies and percentages. Comparison between groups was conducted using the chi-square test. All statistical tests were two sided and *P* < 0.05 was considered as statistically significant. SPSS software was used for all statistical analyses (IBM SPSS statistics for windows, version 24, IBM co., Armonk, NY USA 2015).

## Results

### Priorities of the INDC members

Thirty-two (84%) members of the INDC responded to the first prioritization round. Out of the responders, 56% were men. Average age of the responders was 59.6 ± 7.5 years. Most responders were hospital-based physicians living in central Israel while less than 20% came from the southern and northern periphery. A significant proportion of the council members were community-based practitioners including specialized diabetes nurses and dieticians. The demographic and professional attributes of the INDC members are presented in Table 1. Seven top-ranking priorities are presented in Table 2.

**Table 1:** Socio-demographic characteristics of study populations

		National diabetes council members (n = 32)	Diabetes health professionals (n = 195)	*P*-value
Gender	Women	14 (44%)	130 (67%)	0.01
Age		59.7 ± 7.7	45.8 ± 12	0.02
Area	North	4 (13%)	53 (27%)	0.085
	Center	26 (81%)	117 (60%)	
	South	2 (6%)	25 (13%)	
Profession	Physician	18 (56%)	130 (67%)	< 0.001
	Nurse	5 (16%)	41 (21%)	
	Nutritionist	4 (13%)	24 (12%)	
	Administration	5 (15%)	–	
Place of work	Hospital	18 (57%)	78 (40%)	< 0.001
	Community	11 (33%)	117 (60%)	
	Ministry of Health	3 (10%)	–

**Table 2:** Step 1—Top priorities of the Israeli national diabetes council

Topics	Importance*
Clinics for adults with type 1 diabetes	24
The diabetic foot	21
Treating diabetes in special populations: children and young adults	19
Prevention in at risk/high risk populations (pre-diabetic)	15
Promoting a healthy lifestyle in Israel	14
incentivize an increase in diabetologists and endocrinologists	11
Adapting health care services for diabetes to the economic, social and cultural needs of different population groups	10

*Sum of scores- most important issue was assigned a score of 3, second ranked issue was assigned a score of 2 and the 3rd a score of 1

### Priorities of the hospital and community diabetes professionals

**Fig. 1:**
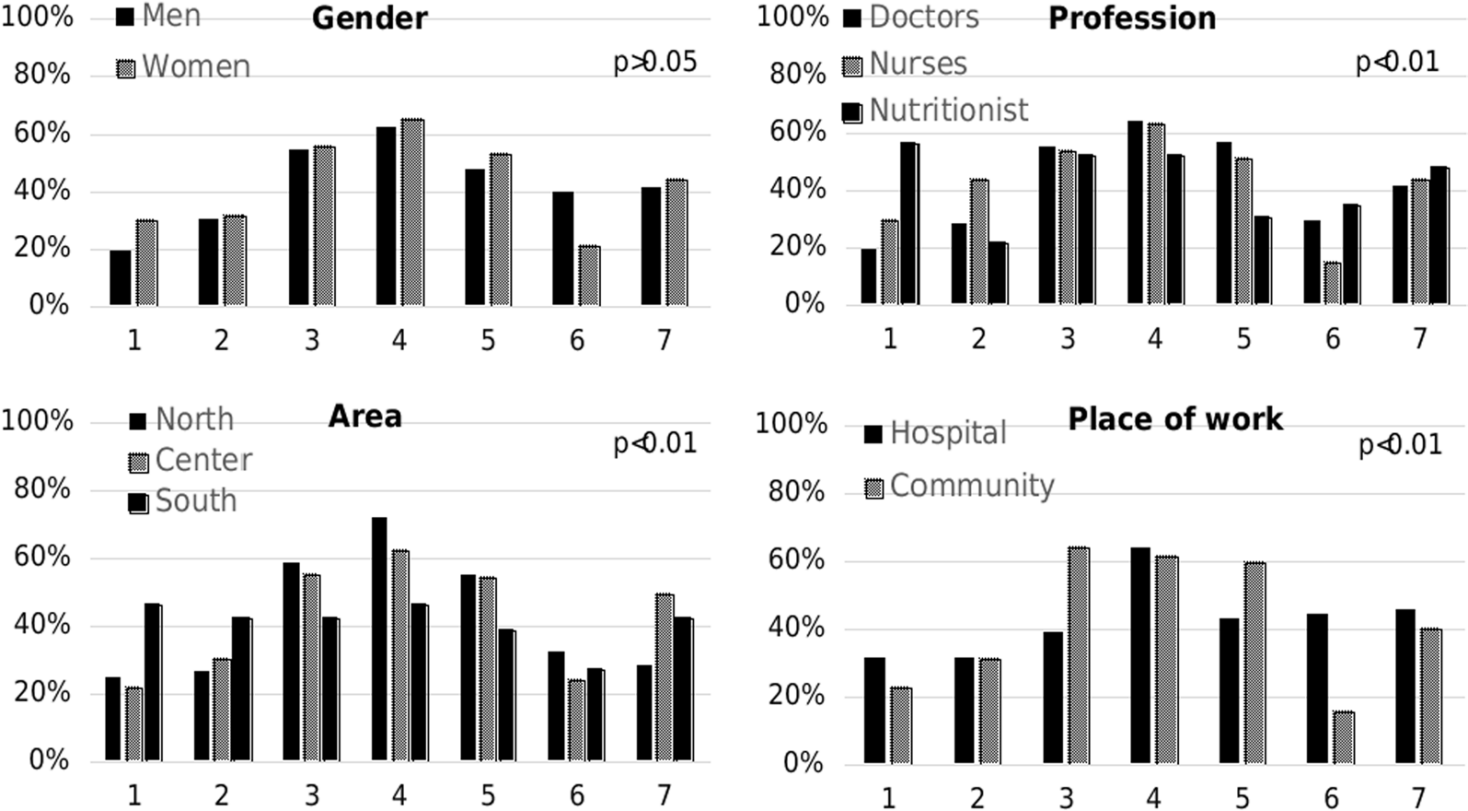
Distribution of priorities of diabetes health professionals by socio-demographic characteristics. 1: Clinics for adults with Type 1 diabetes, 2: The diabetic foot, 3: Treating diabetes in special populations: Children and young adults, 4: Prevention in at risk/high risk populations (pre-diabetic), 5: Promoting a healthy lifestyle in Israel, 6: Incentivize an increase in diabetologists and endocrinologists, 7: Adapting health care services for diabetes to the economic, social and cultural needs of different population groups

One hundred ninety-five responders, 34% males, average age 45.8 + 12 years, participated in step B (characteristics are presented in Table 1). Most responders (60%) worked in community clinics, and 60% live in central Israel. Out of the responders, 67% were physicians and the rest were nursing staff and dieticians. Analysis of priorities according to demographic and professional characteristics revealed several significant differences. Hospital based health professionals chose different seven priorities as compared to community based professionals. Gender and geographical location were not associated with significant different priorities (Fig. 1). As compared to INDC members, higher percentage of health professionals chose the four priorities: “treating diabetes in special populations”, “prevention in high-risk populations”, “promotion of healthy life style”, “Adapting health care services for diabetes to the needs of different population groups” (Table 3).

**Table 3:** Priorities of INDC members and diabetes health professionals

Topics	Council members n = 32	Health professionals n = 195
Clinics for adults with type 1 diabetes	12 (37.5%)	50 (25.6%)
The diabetic foot	10 (31.25%)	60 (30.8%)
Treating diabetes in special populations: children and young adults	11 (34.3%)	106 (54.4%)
Prevention in at risk/high risk populations (prediabetic)	7 (21.8%)	122 (62.6%)
Promoting a healthy lifestyle in Israel	6 (18.7%)	102 (52.3%)
Incentivize an increase in diabetologists and endocrinologists	6 (18.7%)	52 (26.7%)
Adapting health care services for diabetes to the economic, social and cultural needs of different population groups	6 (18.7%)	83 (42.6%)

## Discussion

We demonstrated here for the first time that a concise list of national priorities in the field of diabetes management is attainable. This process is essential having in mind the diabetes related ever-growing needs and expenses in view of restricted public and health resources. We conducted a 2-step priority setting process, which included a small circle of key opinion leaders, members of the national diabetes council, and a much larger nationwide circle of diabetes health professionals. The council members opted mostly for goals focused on specified populations such as clinics for adults with type 1 diabetes, diabetic foot and pediatric and adolescent patients. The top 3 priorities of the larger group of health professionals were mostly in the general field of prevention, namely: high-risk prediabetes population, prevention of obesity and promotion of healthy life-style in Israel. In addition, different priorities were also observed among the health professionals, as community and hospital-based personnel chose different set of priorities. The former group focused on special groups, such as children and young adults as well as life style changes, while the latter one included in their top priorities organizational changes in diabetes services, such as added incentives and more staff positions in endocrinology and diabetology. It is of interest that this different approach was also present in the focused group of physicians. Taken together, these results demonstrate that health professionals and key opinion leaders do not necessary share the same set of priorities and raises the conceptual and provocative question of which priorities should be followed. This question has no simple answer, in particular in the absence of comparable data from other countries. Moreover, there is no strict definition of right and wrong priorities and these decisions are subject to the individual preferences of the responders. It could be argued that the clinical opinion of the health professionals should prevail. However, the key opinion leaders of the national council may be more aware of practical budget restriction and applicability issues and therefore their priorities should be considered. It is also possible that expanding the list of priorities may have eliminate the differences between INDC members and diabetes health professionals by creating a common basis for these two groups. It should be emphasized that diabetes patients are the most important stakeholders in the management of their own disease, and it is mandatory to include a patient reference group. Such approach unrelated to diabetes was reported by Tang and colleagues [3] who surveyed few small communities in southeast Asia. This has several advantages including increasing the knowledge of health policy makers and providing vital information of patients’ individual expectations. However, these surveys are difficult to perform and it is not clear how representative they are regarding larger populations.

Our study has several limitations including the rather small size of the responders’ groups, the restricted number of priorities of only 7 top topics presented to the health professionals and the absences of patients’ priorities. It should be noted that priorities may change with time and social-economic trends and therefore our findings may be time limited. It is also unknown whether our methodology is applicable to other health domains and societies. In addition, the INDC questionnaire was focused on importance and applicability, whereas the health professional’s questionnaire focused only on the importance of priorities. However, it is unlikely that this difference was significant since these two lists were almost identical.

Our study has several strengths. Firstly, it demonstrates for the first time the feasibility of creating a restricted list of top priorities in the field of diabetes management. Secondly and most importantly, if this list is accepted by national health policy makers it may serve as a basis for the future planning of diabetes management in Israel. Last but not least, this approach may be adopted by other national councils in various fields.

## Conclusions

A national prioritization process of action areas in diabetes prevention and care is attainable. The resulting item list is affected by professional considerations. These priorities may be crucial in the implementation of interventions to improve national-level diabetes management. Differences in priorities between the National Council and practicing health professionals, should be addressed in policy making.

## Supplementary Information

**Additional file 1:** List of the 45 priorities included in the national program for diabetes management in Israel.

## Data Availability

The datasets used and/or analyzed during the current study are available from the corresponding author on reasonable request.

## Abbreviations

INDC: Israeli national diabetes council
MOH: Ministry of health
