# Supplementary material for: Establishing priorities for diabetes action goals according to key opinion leaders and health professionals
Source: Isr J Health Policy Res. 2022 Aug 19;11:29. doi: 10.1186/s13584-022-00540-x (PMC9392280; doi:10.1186/s13584-022-00540-x)
Supplement: Supplementary file 1 — Additional file 1: List of the 45 priorities included in the national program for diabetes management in Israel. [file 13584_2022_540_MOESM1_ESM.docx]

### Supplementary material

List of the 45 priorities included in the national program for diabetes management in Israel

| 1 | Promoting a healthy lifestyle in Israel |
| --- | --- |
| 2 | Diabetes prevention in at risk / high risk populations (pre-diabetic) |
| 3 | Diabetes prevention among different social and ethnic groups: Arab society, Ethiopian society, ultra-Orthodox society |
| 4 | Treating diabetes in special populations: Children and young adults |
| 5 | Standardization in performing HbA1c testing |
| 6 | Guidelines for medical monitoring after pregnancy of women who experienced gestational diabetes |
| 7 | Incentivize an increase in diabetologists and endocrinologists |
| 8 | Stratification of Hba1c control target |
| 9 | Balancing diabetes in the community and insulin in family medicine |
| 10 | Balancing diabetes in the hospital |
| 11 | Guidelines for continuity of care between hospitalization and community |
| 12 | Hypoglycemia: Guidelines for identification and preventive intervention |
| 13 | Physical activity in diabetes |
| 14 | Treating risk factors in diabetic |
| 15 | The diabetic foot |
| 16 | Early detection and treatment of diabetic retinopathy |
| 17 | Treating diabetic kidney disease (nephropathy) and preventing kidney failure |
| 18 | Coronary artery disease (CAD) in diabetics – improving diagnosis and treatment |
| 19 | The diabetic brain – brain vascular disease among diabetics |
| 20 | Prevention and treatment of fatty liver disease |
| 21 | Treatment of pain in diabetes |
| 22 | Diabetes care in children and adolescents |
| 23 | Clinics for adults with Type 1 diabetes |
| 24 | Treating diabetes in older adults (geriatrics) |
| 25 | Adapting health care services for diabetes to the economic, social and cultural needs of different population groups |
| 26 | Screening, prevention and care of diabetes in people with severe mental illness |
| 27 | Disabilities and disorders relating to diabetes, in terms of Israel’s National Insurance Institute (NII) |
| 28 | Procedure for issuing driving authorization for diabetic patients |
| 29 | Rights of students with diabetes and standards for aides in schools |
| 30 | Virtual clinic – distance monitoring and support of patients and caregivers via computer |
| 31 | Control of sugar consumption in the Israeli diet |
| 32 | Labeling foods for diabetic patients |
| 33 | Educators for diabetes and other chronic diseases |
| 34 | Internship in diabetes and metabolism in the health professions: Doctors, Nurses, Dieticians |
| 35 | Pharmacological consulting for patients with Type 2 diabetes |
| 36 | Improving compliance with treatment |
| 37 | Training medical staffs on the psychological aspects of diabetes and coping with the disease |
| 38 | Supporting self-care and empowering the patient |
| 39 | Employing digital means to improve compliance with and adherence to diabetes treatment |
| 40 | Patient involvement in the design of services and policy in diabetes |
| 41 | Indices on quality of hospitalization |
| 42 | Registries for prediabetes, diabetes, bariatric surgeries among diabetic patients and gestational diabetes |
| 43 | The cost of diabetes in Israel |
| 44 | National center for diabetes research and policy advancement |
| 45 | Childhood obesity treatment centers |
